# PBAF Subunit Pbrm1 Selectively Influences the Transition from Progenitors to Pre-Myelinating Cells during Oligodendrocyte Development

**DOI:** 10.3390/cells12121556

**Published:** 2023-06-06

**Authors:** Vanessa Waldhauser, Tina Baroti, Franziska Fröb, Michael Wegner

**Affiliations:** Institut für Biochemie, Friedrich-Alexander-Universität Erlangen-Nürnberg, Fahrstrasse 17, D-91054 Erlangen, Germany; vanessa.polanetzki@fau.de (V.W.); tina.baroti@fau.de (T.B.); franziska.froeb@fau.de (F.F.)

**Keywords:** glia, myelin, oligodendrocyte, chromatin remodeling, polybromo-1

## Abstract

Oligodendrocyte development is accompanied by defined changes in the state of chromatin that are brought about by chromatin remodeling complexes. Many such remodeling complexes exist, but only a few have been studied for their impact on oligodendrocytes as the myelin-forming cells of the central nervous system. To define the role of the PBAF remodeling complex, we focused on Pbrm1 as an essential subunit of the PBAF complex and specifically deleted it in the oligodendrocyte lineage at different times of development in the mouse. Deletion in late oligodendrocyte progenitor cells did not lead to substantial changes in the ensuing differentiation and myelination processes. However, when Pbrm1 loss had already occurred in oligodendrocyte progenitor cells shortly after their specification, fewer cells entered the pre-myelinating state. The reduction in pre-myelinating cells later translated into a comparable reduction in myelinating oligodendrocytes. We conclude that Pbrm1 and, by inference, the activity of the PBAF complex is specifically required at the transition from oligodendrocyte progenitor to pre-myelinating oligodendrocyte and ensures the generation of normal numbers of myelinating oligodendrocytes.

## 1. Introduction

Oligodendroglial cells are the myelin-forming glia of the central nervous system (CNS) and are required for rapid saltatory conduction and fast information processing. They develop from oligodendrocyte progenitor cells (OPCs), which are themselves derived from neuroepithelial precursor cells of the ventricular zone [1]. During their development, substantial changes in gene expression occur that require adaptations in the gene regulatory network, as well as alterations in chromatin structure. Whereas much is already known about the transcription factors that act as major determinants of the regulatory network, less is known about the chromatin remodeling complexes that shape the chromatin landscape [2,3].

Thus far, the impact of the BAF, the Ep400/Tip60 and the Chd7/8-containing complexes has been investigated during oligodendrogenesis [4,5,6,7,8,9]. All studies followed a similar approach and deleted the respective ATP-hydrolyzing, energy-generating subunit, as the complex is unstable without this subunit and is inactive. In the various investigations, complex-specific roles in specification, lineage progression and/or terminal differentiation were defined [4,5,6,7,8,9].

Another important chromatin remodeling complex that has not yet been studied in oligodendroglial development is the PBAF complex. In other cell types and developmental processes, the PBAF complex has been shown to regulate cell-type identity and cell differentiation [10,11,12,13]. The PBAF complex shares its ATP-hydrolyzing subunit Brg1 with a fraction of the BAF complexes. Whereas Brg1 is not specific to the PBAF complex, several specific signature subunits are. These are also required for the stability and functioning of the PBAF complex and one of them is Pbrm1, also known as polybromo-1, PB1 or BAF180 [14].

Pbrm1 has been linked to transcriptional processes—in particular, to ligand-activated transcription and nuclear receptor function [11,15]. Additionally, it appears to participate in sister chromatid cohesion and in the repriming of stalled replication forks [16,17]. Pbrm1 contains multiple consecutive bromodomains and an additional HMG domain. It is the PBAF subunit that is most frequently mutated in human cancers, with a particularly close association with primary clear cell renal cell carcinoma [14]. Considering its central role within the PBAF complex, we decided to delete Pbrm1 at specific times of oligodendrocyte development and hypothesized that such an approach would not only unravel Pbrm1’s but also PBAF’s functions in oligodendroglial cells.

## 2. Materials and Methods

### 2.1. Husbandry and Breeding of Mice

Mice carrying floxed *Pbrm1* alleles (strain B6;129-Pbrm1tm1Zhwa/J) [18] were obtained from the Jackson Laboratories (Bar Harbor, ME, USA) and were bred with mice expressing a *Cnp1^Cre^* allele [19] or a *Sox10::Cre* Bac transgene [20]. PCR genotyping was as described [19,20,21]. All mice were on a mixed C3H x C57Bl/6J background and kept under standard housing conditions, including 12:12 h light–dark cycles and continuous access to food and water. Timed matings were set up to generate litters at embryonic day (E) 16.5 and at postnatal days (P) 0, 7, 14, 21. Spinal cord and forebrain tissue was dissected from both sexes and processed for histological, immunohistochemical and in situ hybridization studies [22].

### 2.2. Immunohistochemical Analysis and In Situ Hybridization

Spinal cord and forebrain tissue underwent fixation in 4% paraformaldehyde, cryoprotection in 30% sucrose, embedding in Tissue Freezing Medium (Leica, Wetzlar, Germany) and cryotome sectioning at 10 µm thickness, before staining with the following primary antibodies: anti-Olig2 mouse antibody (Millipore, Darmstadt, Germany, #3756534, 1:1000), anti-Olig2 rabbit antibody (Millipore, #AB9610, 1:1000 dilution), anti-Bcas1 rabbit antibody (Synaptic System, Göttingen, Germany, #445-003, 1:1000 dilution), anti-Myrf rabbit antibody (self-made, 1:1000 dilution) [23], anti-Iba1 rabbit antibody (Wako, Osaka, Japan, #019-19741, 1:250 dilution), anti-cleaved caspase 3 rabbit antibody (Cell Signaling Technology, Danvers, MA, USA #9661, 1:200 dilution), anti-Mcm2 rabbit antibody (Cell Signaling Technology, #4007, 1:100 dilution), anti-Pdgfra rabbit antibody (Santa Cruz, Dallas, TX, USA, #E-1210, 1:300 dilution), anti-Pdgfra goat antibody (R&D Systems, Minneapolis, MN, USA, #AF1062, 1:100 dilution), anti-Sox10 goat antibody (1:3000 dilution) [24], anti-Sox10 guinea pig antibody (self-made, 1:1000 dilution) [25], anti-Sox6 guinea pig antibody (self-made, 1:1000 dilution) [26], anti-Pbrm1 guinea pig antibody (self-made, 1:5000 dilution) [21], anti-Ki67 rat antibody (Invitrogen, Waltham, MA, USA, #14-5698-82, 1:500 dilution) and anti-MBP rat monoclonal antibody (Bio-Rad, Hercules, CA, USA, #MCA409S, 1:500 dilution). For fluorescent labeling, Cy3-, Cy5- or Alexa488-coupled secondary antibodies were used (Dianova, Hamburg, Germany, # 705-175-14, 705-165-147, 706-165-148, 706-175-148, 706-545-148, 711-165-152, Invitrogen # A11055 and A21206, Jackson ImmunoResearch Labs (West Grove, PA, USA) #711-175-152, at 1:200 to 1:500 dilution). Nuclei were counterstained with 4′,6-diamidino-2-phenylindole (Dapi, Sigma, St. Louis, MO, USA, #D9542). The ApopTag Red In Situ Apoptosis Detection Kit was used for TUNEL assays, according to the manufacturer’s protocol (Chemicon, Temecula, CA, USA). For in situ hybridization, digoxigenin-labeled *Plp1* or *Mag* antisense riboprobes were used [27]. Acquisition of fluorescence images was performed with a DMI 6000B inverted microscope (Leica) coupled to a DFC 360FX camera (Leica). For quantifications, tissue from three individuals was analyzed per genotype and used to determine the mean. Error bars represent the standard error of the mean (SEM). Per tissue sample, fluorescent signals were counted for each marker in images of three separate 10-µm-thick transverse sections and averaged. For spinal cord images, all signals were counted so that numbers corresponded to marker-positive cells per imaged section. For forebrain images, all signals within the corpus callosum were counted, and numbers corresponded to marker-positive cells per mm^2^ of callosal section. Values for the nine counted sections per marker and genotype conformed to a Gaussian distribution.

### 2.3. Statistical Analysis

Whenever possible, experiments and analyses were carried out in a blinded fashion. No sample calculation was performed. Results from individual mice were treated as biological replicates. To determine whether differences in cell numbers or amounts were statistically significant (*, *p* ≤ 0.05; **, *p* ≤ 0.01, ***, *p* ≤ 0.001), an unpaired two-tailed Student’s *t* test was performed, in which matching data sets (i.e., from controls and conditional Pbrm1 mutant mice at a particular age) were compared using GraphPad Prism 8 (GraphPad software, La Jolla, CA, USA).

## 3. Results

### 3.1. Pbrm1 Is Present in Oligodendroglial Cells throughout Their Development

To investigate whether and when the Pbrm1 protein is present in oligodendroglial cells throughout their development, co-immunohistochemical studies were carried out on spinal cord sections of wildtype mouse embryos and pups.

**Figure 1:**
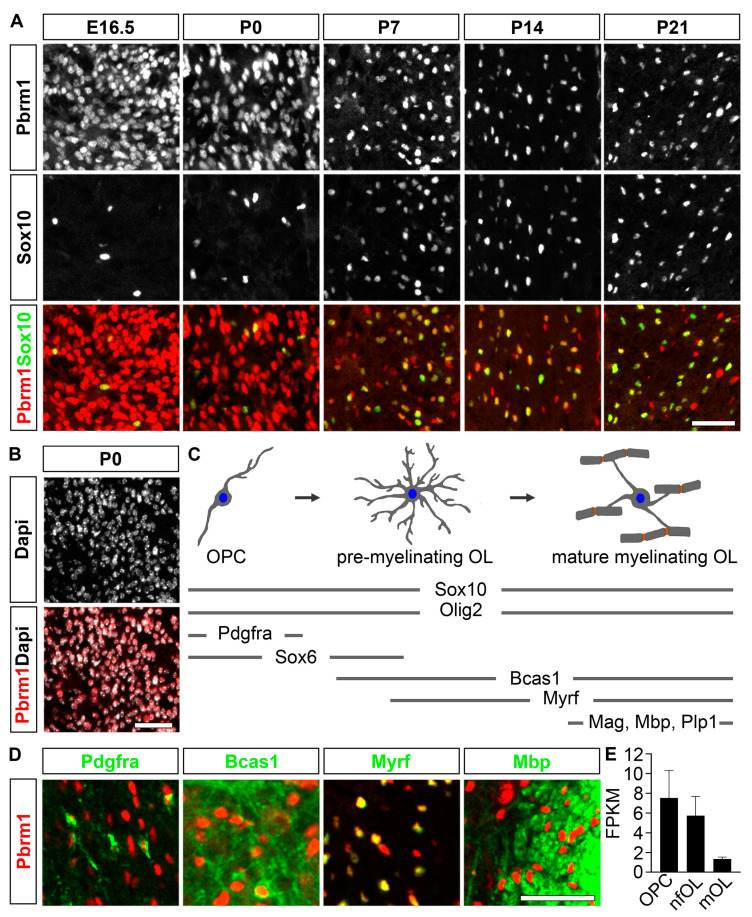
Pbrm1 is expressed throughout oligodendroglial development in the pre- and postnatal spinal cord. (**A**) Co-immunohistochemical staining for Pbrm1 (red) and Sox10 (green) in spinal cord sections at E16.5, P0, P7, P14, P21. (**B**) Co-staining for Pbrm1 (red) and Dapi (white) in spinal cord sections at P0. (**C**) Schematic representation of oligodendroglial development (upper half) from OPC stage via pre-myelinating to myelinating oligodendrocyte (OL), with periods of expression indicated for several markers (lower half). (**D**) Co-localization of Pbrm1 (red) with Pdgfra as OPC marker, Bcas1 and Myrf as markers for (pre-) myelinating oligodendrocytes and Mbp as myelin protein by antibody co-staining. Scale bars: 100 µm (**A**,**B**), 50 µm (**D**). (**E**) Expression levels of *Pbrm1* in OPCs and newly formed (nf) and myelinating (m) oligodendrocytes in fragments per kilobase of exon per million mapped fragments (FPKM) according to https://www.brainrnaseq.org/ (accessed on 12 April 2023).

These revealed the continuous presence of Pbrm1 in Sox10-positive oligodendroglial cells from E16.5 (when most cells are still in the OPC stage) to P21 (when most have already entered the myelinating stage) (Figure 1A). They also showed that Pbrm1 is likewise expressed in most (if not all) Dapi-stained nuclei within the spinal cord (Figure 1B), arguing that Pbrm1 occurrence within the CNS is not restricted to oligodendroglial cells. Astrocytes and neurons also contain the protein.

A closer analysis of Pbrm1 expression in cells of the oligodendroglial lineage revealed that Pbrm1 co-localized with Pdgfra as a marker of the OPC state, as well as with Bcas1 and Myrf as markers of pre-myelinating and myelinating stages and with the myelin protein Mbp (Figure 1C,D). Pbrm1 expression throughout oligodendroglial development was also confirmed at a transcript level. A search of publicly available datasets such as Brain RNA-Seq (www.brainrnaseq.org, accessed on 12 April 2023) revealed the presence of *Pbrm1* transcripts in OPCs as well as newly formed and myelinating oligodendrocytes, with transcript levels decreasing as development proceeds (Figure 1E).

### 3.2. Pbrm1 Deletion in Late OPCs Fails to Impact Subsequent Oligodendrocyte Development

**Figure 2:**
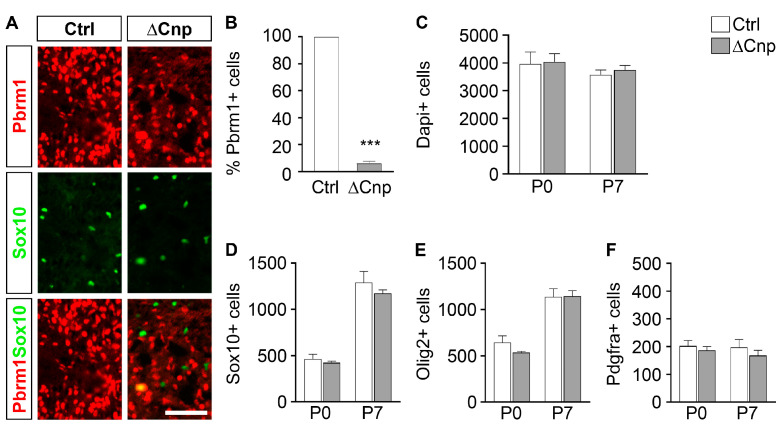
Pbrm1 deletion in late spinal cord OPCs is efficient and has no effects on total oligodendroglial cell and OPC numbers. (**A**) Immunohistochemical staining of transverse spinal cord sections of newborn control (Ctrl) and Pbrm1∆Cnp (∆Cnp) mice using antibodies directed against Pbrm1 (red) and Sox10 (green). Scale bar: 100 µm. (**B**) Determination of the percentage of Pbrm1-positive cells among all oligodendroglial cells in the spinal cords of control (white bars) and Pbrm1∆Cnp (grey bars) mice at P0. (**C**) Quantification of overall cell numbers in spinal cord sections of Pbrm1∆Cnp and control mice at P0 and P7 from Dapi signals. (**D**–**F**) Determination of the absolute number of Sox10- (**D**) or Olig2-positive (**E**) oligodendroglial cells or Pdgfra-positive OPCs (**F**) per spinal cord section in both genotypes at P0 and P7. Data represent mean ± SEM (*n* = 3). Differences from control were statistically significant as indicated (Student’s *t* test; ***, *p* ≤ 0.001).

By combining the *Pbrm1^fl^* allele [18] with the *Cnp1^Cre^* allele [19], we specifically targeted the floxed *Pbrm1* allele in oligodendroglial cells of the spinal cord in their late OPC stage. Co-immunohistochemical staining and quantification revealed that Pbrm1 was effectively deleted in the vast majority of oligodendroglial cells by P0, with only 6.3 ± 1.5% of all Sox10-expressing cells still positive for the protein (Figure 2A,B). At the same time, Pbrm1 deletion in the spinal cord was restricted to the oligodendroglial population.

When counting overall cell numbers per spinal cord section in these Pbrm1∆Cnp mice, we did not detect any statistical difference from controls either at P0 or at P7 (Figure 2C). Similarly, the total number of oligodendroglial cells was comparable between both genotypes, independent of whether cells were identified by Sox10 or Olig2 as a lineage marker (Figure 2D,E).

Stage-specific markers allowed a closer examination of single developmental stages and the distribution of oligodendroglial cells between them. By using Pdgfra, we first determined that OPC numbers were not altered in the spinal cords of Pbrm1∆Cnp mice (Figure 2F). This was expected as Pbrm1 deletion occurs in late-stage OPCs and should not affect their development.

**Figure 3:**
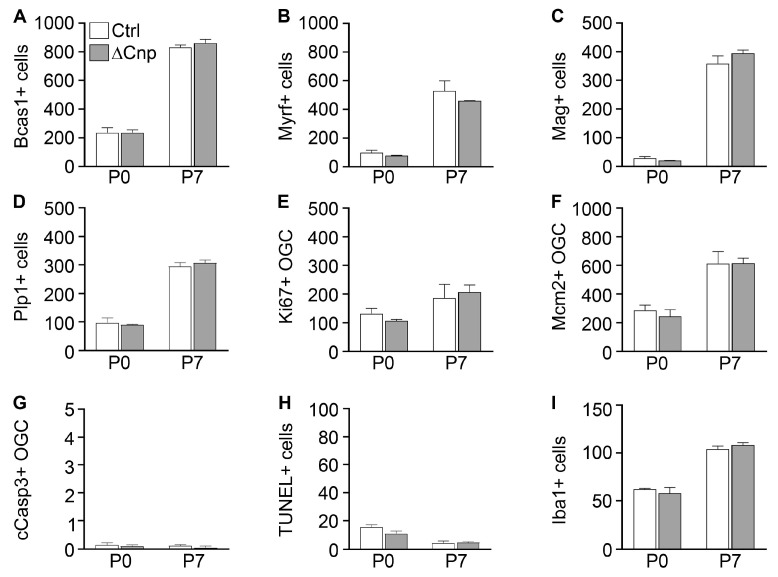
Pbrm1 deletion in late spinal cord OPCs has no effects on oligodendroglial proliferation, cell death and differentiation. (**A**–**I**) Determination of the absolute number of Bcas1 (**A**), Myrf (**B**), *Mag* (**C**) and *Plp1* (**D**) expressing cells, the Ki67- (**E**), Mcm2- (**F**) and cleaved caspase 3- (cCasp3, (**G**)) positive oligodendroglial cells (OGC), TUNEL signals (**H**) or Iba1-positive microglia (**I**) per spinal cord section in control (Ctrl, white bars) and Pbrm1∆Cnp (∆Cnp, grey bars) mice at P0 and P7. Data were obtained from immunohistochemical stainings (**A**,**B**,**E**–**I**) and in situ hybridizations (**C**,**D**) and represent mean ± SEM (*n* = 3). No statistically significant differences were obtained.

When cells leave the OPC stage and become pre-myelinating oligodendrocytes, cells activate Bcas1 and—with a slight delay—Myrf expression (Figure 1C). Once induced, Bcas1 and Myrf remain expressed in myelinating oligodendrocytes. As a consequence, these markers are capable of detecting oligodendroglial cells from the pre-myelinating stage onwards. Quantification of both Bcas1 and Myrf failed to reveal any significant differences in cell numbers between Pbrm1∆Cnp and control mice at P0 and P7 (Figure 3A,B). Similar cell numbers between both genotypes were also obtained when analysis was restricted to myelin-gene-expressing cells by using *Mag* or *Plp1* as markers (Figure 3C,D).

In line with these observations, we did not detect any differences in proliferation among oligodendroglial cells using either Ki67 or Mcm2 as a marker (Figure 3E,F). Cell survival was also comparable between the genotypes at both time points of analysis, as the TUNEL analysis or staining for cleaved caspase 3 did not yield any evidence of altered cell death rates (Figure 3G,H). There were no signs of inflammation or microgliosis from Iba1 stainings (Figure 3I). We conclude from these data that Pbrm1 appears dispensable for oligodendroglial development from the pre-myelinating stage onwards.

### 3.3. Pbrm1 Deletion in OPCs Soon after Specification Reduces the Numbers of Myelinating and Total Oligodendroglial Cells

**Figure 4:**
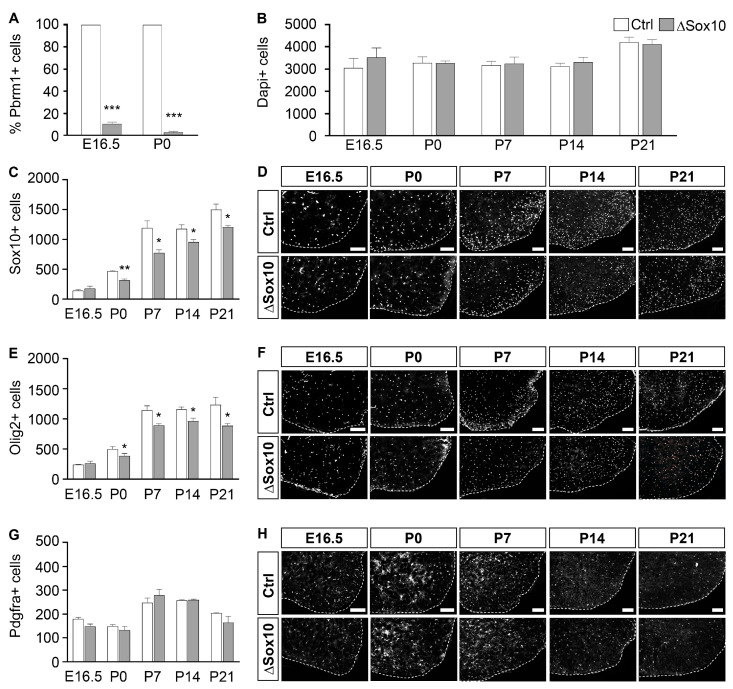
Pbrm1 deletion in early spinal cord OPCs is efficient and affects oligodendroglial cell numbers. (**A**) Determination of the percentage of Pbrm1-positive cells among all oligodendroglial cells in the spinal cords of control (Ctrl, white bars) and Pbrm1∆Sox10 (∆Sox10, grey bars) mice at E16.5 and P0. (**B**) Quantification of overall cell numbers in spinal cord sections of control and Pbrm1∆Sox10 mice from E16.5 to P21 from Dapi signals. (**C**–**H**) Determination of the absolute number of Sox10- (**C**), Olig2- (**E**) and Pdgfra-positive (**G**) oligodendroglial cells per spinal cord section in control and Pbrm1∆Sox10 mice at E16.5 and during the first three postnatal weeks. Data represent mean ± SEM (*n* = 3). They were obtained from immunohistochemical stainings as shown in representative images (ventral right quarter, marked by dotted line and placed on black background) for Sox10 in (**D**), Olig2 in (**F**) and Pdgfra in (**H**). Scale bars: 100 µm. Differences from control were statistically significant as indicated (Student’s *t* test; *, *p* ≤ 0.05; **, *p* ≤ 0.01; ***, *p* ≤ 0.001).

To extend our analysis to earlier phases of oligodendroglial cells, we exchanged the Cre to be combined with the *Pbrm1^fl^* allele and replaced the *Cnp1^Cre^* allele with a *Sox10::Cre* encoded on a BAC transgene [20]. In the resulting Pbrm1∆Sox10 mice, Cre recombinase set in early after the specification event in OPCs, as previously shown [26]. As a consequence, Pbrm1 was deleted in 89.2 ± 1.5% of all oligodendroglial cells at E16.5 and in 96.9 ± 0.9% at P0 and remained restricted to the oligodendroglial lineage (Figure 4A).

Again, the counting of cell numbers in the spinal cord from Dapi signals failed to reveal significant alterations in Pbrm1∆Sox10 mice as compared to controls over the whole period of analysis from E16.5 to P21 (Figure 4B). Overall numbers of Sox10- or Olig2-positive oligodendroglial cells were also initially comparable at E16.5, but became lower in Pbrm1∆Sox10 mice at P0 by approximately 23–30% (Figure 4C–F). This reduction remained stable throughout the first three postnatal weeks (i.e., 20–28% at P21 depending on marker). Interestingly, it did not affect the Pdgfra-positive OPC population. Their numbers remained similar in both genotypes at all times analyzed (Figure 4G,H).

**Figure 5:**
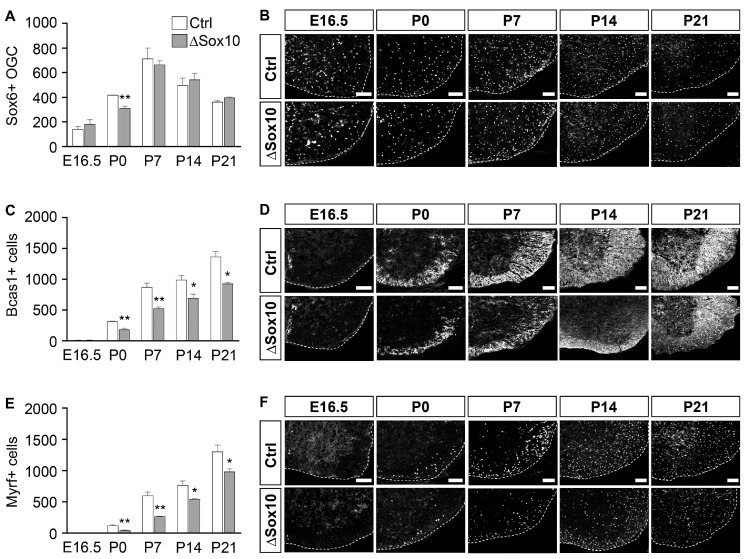
Pbrm1 deletion in early OPCs affects numbers of pre-myelinating oligodendrocytes in the spinal cord. (**A**–**F**) Determination of the absolute number of Sox6- (**A**), Bcas1- (**C**) and Myrf-positive (**E**) oligodendroglial cells per spinal cord section in control (Ctrl, white bars) and Pbrm1∆Sox10 (∆Sox10, grey bars) mice at E16.5 and during the first three postnatal weeks. Data represent mean ± SEM (*n* = 3). They were obtained from immunohistochemical stainings. Representative spinal cord images (ventral right quarter, marked by dotted line and placed on black background) are shown for Sox6 in (**B**), Bcas1 in (**D**) and Myrf in (**F**). Scale bar: 100 µm. Differences from control were statistically significant as indicated (Student’s *t* test; *, *p* ≤ 0.05; **, *p* ≤ 0.01).

As with Pdgfra, Sox6 is present in OPCs but remains expressed in early pre-myelinating oligodendrocytes, before being terminated as these cells continue their development into myelinating cells [25,28]. In line with this expression pattern, Sox6-positive cell numbers in the spinal cord were comparable between Pbrm1∆Sox10 and control mice at E16.5, when most oligodendroglia were still in the OPC stage (Figure 5A,B). With many more oligodendroglial cells in the pre-myelinating stage at P0, a significant difference in Sox6-positive cells became apparent, with numbers being approximately 25% lower in Pbrm1∆Sox10 mice than in controls. By P14, the difference in the two genotypes had disappeared, as many oligodendroglial cells had progressed from the pre-myelinating into the myelinating stage, so that they were no longer Sox6-positive.

The expression of Bcas1 or Myrf starts in pre-myelinating oligodendrocytes and continues in myelinating ones [29,30]. When they were used as markers, we again failed to detect significant differences between genotypes at E16.5 (Figure 5C–F). However, substantial reductions were visible in the numbers of Bcas1- or Myrf-positive cells in the Pbrm1∆Sox10 mice by P0 (41% for Bcas1 and 71% for Myrf) and P7 (40% for Bcas1 and 55% for Myrf). In absolute numbers, the decrease in Bcas1- and Myrf-positive cells at P7 was comparable to the one observed for all oligodendroglial cells. This suggests that Pbrm1 influences oligodendroglial development at the transition from the OPC into the pre-myelinating stage.

The lower levels of pre-myelinating cells also translated into reduced numbers of myelinating oligodendrocytes. This was already apparent from the number of Bcas1- or Myrf-positive cells after P14, when both markers predominantly labeled myelinating cells (Figure 5C–F).

**Figure 6:**
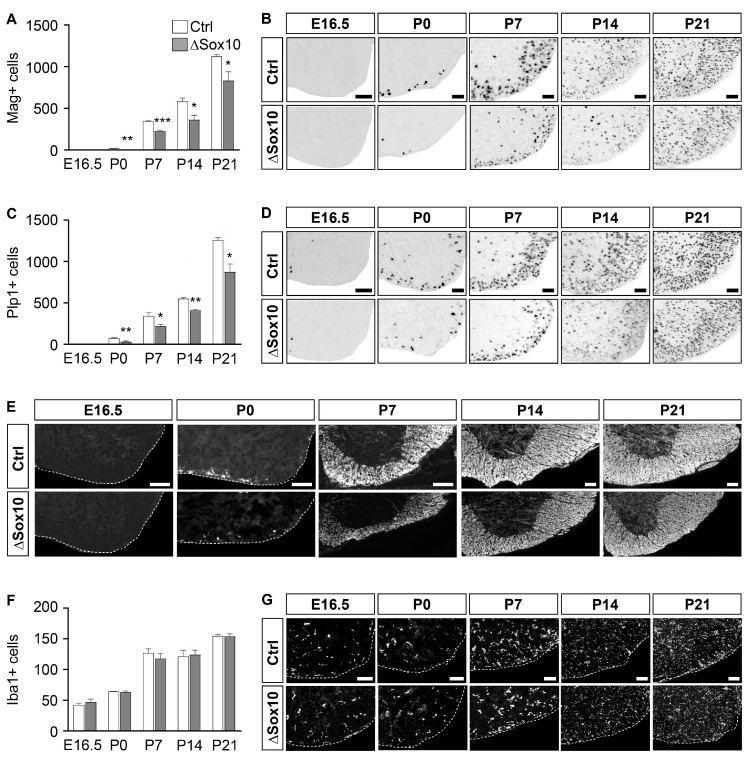
Pbrm1 deletion in early OPCs affects numbers of myelinating oligodendrocytes in the spinal cord. (**A**–**D**) Determination of the absolute number of *Mag*- (**A**) and *Plp1*-positive (**C**) oligodendroglial cells per spinal cord section in control (Ctrl, white bars) and Pbrm1∆Sox10 (∆Sox10, grey bars) mice at E16.5 and during the first three postnatal weeks, as obtained from in situ hybridizations. Representative spinal cord images (ventral right quarter, placed on white background) are shown for *Mag* in (**B**) and *Plp1* in (**D**). (**E**) Visualization of myelin by Mbp immunohistochemistry in control and Pbrm1∆Sox10 mice at indicated times. (**F**,**G**) Determination of the absolute number of Iba1-positive (**F**) microglia per spinal cord section in control and Pbrm1∆Sox10 mice as obtained from immunohistochemical stainings. Representative spinal cord images in (**E**,**G**) show the ventral right quarter, marked by dotted line and placed on a black background. Scale bars: 100 µm. Data represent mean ± SEM (*n* = 3). Differences from control were statistically significant as indicated (Student’s *t* test; *, *p* ≤ 0.05; **, *p* ≤ 0.01; ***, *p* ≤ 0.001).

Additional confirmation for this finding came from in situ hybridizations for *Mag* and *Plp1* (Figure 6A–D). Cells positive for *Mag* or *Plp1* were substantially fewer in Pbrm1∆Sox10 mice at P0, when they became detectable. By P21, the difference in *Mag*- or *Plp1*-positive oligodendrocytes between genotypes roughly matched the overall difference in oligodendroglial cells. This 26–31% difference in myelinating cells at P21 was, however, difficult to detect by Mbp immunostaining (Figure 6E). Differences in Mbp signal intensities were more obvious at P0 and P7. Staining of microglia with Iba1 as a marker did not yield any signs of an inflammatory response to the reduced number of pre-myelinating and myelinating oligodendrocytes (Figure 6F,G).

**Figure 7:**
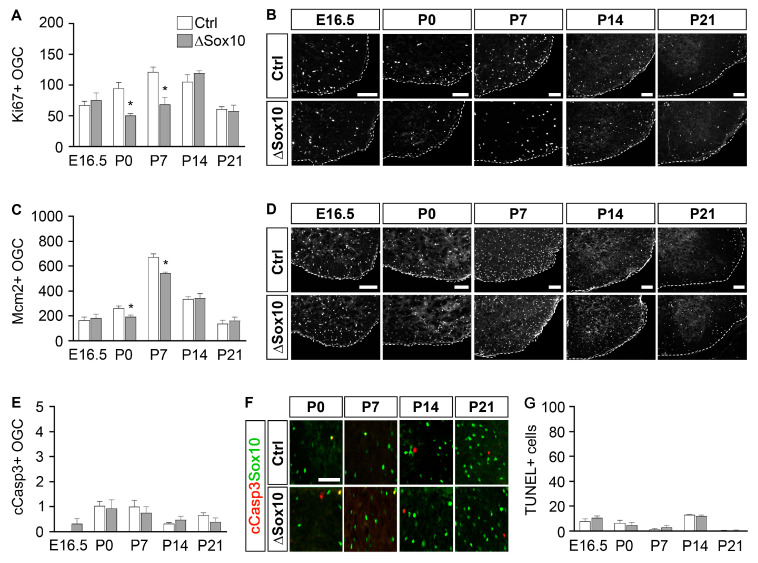
Pbrm1 deletion in early OPCs affects oligodendroglial proliferation in the spinal cord, but not survival. (**A**–**F**) Determination of the absolute number of Ki67 (**A**) and Mcm2 (**C**) expressing oligodendroglial cells (OGC), the number of cleaved caspase 3-positive OGC (cCasp3-, (**E**)) and TUNEL signals (**G**) per spinal cord section in control (Ctrl, white bars) and Pbrm1∆Sox10 (∆Sox10, grey bars) mice at E16.5 and during the first three postnatal weeks, as obtained from immunohistochemical stainings (*n* = 3). Representative images for Ki67 in (**B**) and for Mcm2 in (**D**) show the ventral right quarter, marked by dotted line and placed on a black background. Representative images for cCasp3 (red) are higher white matter magnifications (**F**), with Sox10-positive cells (green) additionally shown. Scale bars: 100 µm. Data represent mean ± SEM (*n* = 3). Differences from control were statistically significant as indicated (Student’s *t* test; *, *p* ≤ 0.05).

When proliferation was studied using either Ki67 or Mcm2 as a marker, we observed lower numbers of proliferating oligodendroglial cells in the spinal cord tissue of Pbrm1∆Sox10 mice as compared to controls selectively at P0 and P7 (Figure 7A–D). At all other time points analyzed (i.e., E16.5, P14 and P21), no differences were detected. In contrast to the transient alterations in proliferation, we did not notice substantial alterations in cell death throughout the entire period of analysis, from E16.5 to P21, when the spinal cords of Pbrm1∆Sox10 mice were compared to controls using either cleaved caspase 3 or TUNEL as a marker (Figure 7E–G).

**Figure 8:**
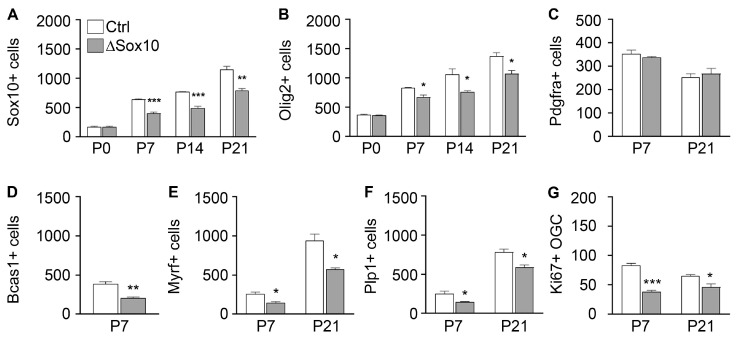
Pbrm1 deletion in early OPCs affects oligodendroglial development in the forebrain. (**A**–**G**) Determination of the absolute number of Sox10- (**A**), Olig2- (**B**), Pdgfra- (**C**), Bcas1- (**D**), Myrf- (**E**), *Plp1*- (**F**) and Ki67-positive (**G**) oligodendroglial cells (OGC) per mm^2^ in the corpus callosum of control (Ctrl, white bars) and Pbrm1∆Sox10 (∆Sox10, grey bars) mice during the first three postnatal weeks, as determined from immunohistochemical stainings (**A**–**E**,**G**) and in situ hybridizations (**F**). Data represent mean ± SEM (*n* = 3). Differences from control were statistically significant as indicated (Student’s *t* test; *, *p* ≤ 0.05; **, *p* ≤ 0.01; ***, *p* ≤ 0.001). Note that single cells were not resolved with Bcas1 at P21 so that cell numbers could not be determined.

To assess the validity of our findings in the spinal cord for other CNS regions, we repeated key aspects of our analysis in the forebrains of Pbrm1∆Sox10 mice and studied oligodendroglial cells in the corpus callosum as a major white matter tract. We observed a significant decrease in oligodendroglial cells in Pbrm1∆Sox10 mice with both Sox10 and Olig2 as markers from P7 onwards (Figure 8A,B). While there was no difference in the number of Pdgfra-positive OPCs between control and Pbrm1∆Sox10 mice at either P7 or P21 (Figure 8C), markers for pre-myelinating and myelinating oligodendrocytes pointed to a substantial reduction in these populations at P7 and P21 (Figure 8D–F). As previously observed in the spinal cord, lower oligodendroglial cell numbers in the corpus callosum of Pbrm1∆Sox10 mice correlated with reduced proliferation rates (Figure 8G). Taking into account that oligodendroglial development in the forebrain is delayed by approximately one week relative to the spinal cord, results in both CNS regions correlate well.

## 4. Discussion

In this manuscript, we have shown that the deletion of Pbrm1 immediately after specification did not affect the number or distribution of OPCs in the spinal cord, but led to a significant decrease in myelinating oligodendrocytes. A more detailed stage-specific analysis of oligodendroglial development furthermore indicated that the loss of Pbrm1 primarily affected the transition from OPC to pre-myelinating oligodendrocytes and probably less the ensuing final developmental steps, including myelination. Similar results were obtained in the forebrain.

A lack of impact on the myelination process itself was also indicated by our failure to observe phenotypic alterations in oligodendrocytes when Pbrm1 deletion occurred in late OPCs under the control of a *Cnp1^Cre^* allele. This may indicate that *Cnp1^Cre^*-mediated deletion occurs too late to interfere with Pbrm1 function during the transition from OPC to pre-myelinating oligodendrocyte, probably because the Pbrm1-dependent chromatin remodeling events required for the transition are already in place.

Involvement in the early phases of oligodendroglial differentiation is not the only function reported for Pbrm1 in cellular differentiation processes. It has, for instance, been shown that Pbrm1 has essential roles in trophoblast, heart and kidney proximal tubular cell development [12,13]. Interestingly, however, Pbrm1 has little impact on Schwann cell development in the peripheral nervous system [21]. This complements several previous findings that point to substantial differences in the components and organization of the gene regulatory network in oligodendroglial cells and Schwann cells compared to their myelin-producing counterparts in the peripheral nervous system [31]. It furthermore argues that myelin in the central and peripheral nervous systems is likely a result of convergent evolution.

Considering the fact that Pbrm1 is an essential subunit of the PBAF complex, it appears reasonable to assume that functions attributed to Pbrm1 will require activity of the PBAF complex. As the PBAF complex shares several subunits with the BAF complex, one may consider how the activities of both complexes compare in a specific developmental process. For oligodendroglial development, an answer is at least partially possible. Previous studies examined at the BAF complex in oligodendroglial cells by deleting the central energy-generating Brg1 subunit [4,9]. Depending on the Cre allele used and the resulting timing of Brg1 deletion, these studies pointed to an essential requirement of Brg1 for oligodendroglial specification and additional roles in terminal differentiation [4,9]. It is, however, not straightforward to extrapolate from Brg1 to BAF complex functions. This is because not all BAF complexes contain Brg1 as an ATP-hydrolyzing subunit but, alternatively, the related Brm [14], which is expressed at comparable levels to Brg1 during oligodendroglial development (see www.brainseq.org, accessed on 12 April 2023). An additional complication results from the fact that Brg1 is not only present in BAF complexes but is similarly active in PBAF complexes. This highlights the possibility that previously identified Brg1 functions are partly or fully associated with PBAF instead of BAF complexes.

The current study helps to attribute functions to BAF and PBAF complexes in at least one aspect. We previously showed a mild myelination deficit in mice carrying a combination of *Brg1^fl^* and *Cnp1^Cre^* alleles [4]. Considering the fact that mice with a combination of *Pbrm1^fl^* and *Cnp1^Cre^* alleles in the present study did not exhibit a comparable phenotype, it appears reasonable to assume that the Brg1-related myelination defect can be attributed to BAF and not to PBAF complex activity.

Our current study does not provide a molecular mechanism for the role of Pbrm1 in the transition of OPCs to pre-myelinating oligodendrocytes. The observed transient reduction in OPC proliferation at the time of the massive conversion of OPCs into pre-myelinating oligodendrocytes may point to the fact that Pbrm1 exerts its effects during the final round of OPC division, either by altering the timing or the mode of division. In this context, it is worth mentioning that Pbrm1 plays a critical role in sister chromatid cohesion [17] and that PBAF complexes are highly enriched at the kinetochores of mitotic chromosomes [32]. Alternatively, the close association of Pbrm1 and PBAF complexes with nuclear receptor function and ligand-activated transcription [15] may be relevant in this context, as several nuclear receptor ligands and thyroid hormones have strong differentiation-promoting effects on oligodendroglial cells [33,34]. Future studies will help to resolve this issue and clarify which of the known molecular activities of Pbrm1 and the PBAF complex are relevant in the context of oligodendrocyte differentiation.

## Data Availability

All data generated and analyzed during this study are included in this article.
